# Robot-Assisted Laparoscopic Radical Prostatectomy: Technique and Outcomes of 700 Cases

**Published:** 2009-09

**Authors:** John R. Carlucci, Fatima Nabizada-Pace, David B. Samadi

**Affiliations:** *Department of Urology, Mount Sinai School of Medicine, New York, USA*

**Keywords:** robotic prostatectomy, DaVinci prostatectomy, radical prostatectomy, prostate cancer

## Abstract

**Background:**

Robotic prostatectomy techniques are evolving rapidly as the procedure gains popularity and continues to be compared to the gold standard of open retropubic radical prostatectomy. Our objective is to report the operative technique and outcomes of 700 consecutive robotic radical prostatectomies performed by a single surgeon at Mount Sinai Medical Center between May 2007 and October 2008. Data was prospectively collected in an Internal Review Board (IRB)-approved database.

**Surgical Procedure:**

Key aspects of our technique include 1) dissection of the bladder neck first; 2) minimal to no use of cautery from posterior bladder neck dissection onward; 3) leaving endopelvic fascia intact until after neurovascular bundles dissected; 4) preservation of a wide margin of endopelvic fascia; 5) posterior dissection and nerve-sparing in a medial to lateral direction; 6) cold transection of the dorsal venous complex without prior ligation; and 7) posterior bladder neck reconstruction.

**Results:**

Mean OR time from skin incision to skin closure was 124 minutes [48-266]; mean robotic time was 88 minutes [36−190]. Mean EBL was 69.3ml [5−400]. Mean and median length of stay was 1 day. Overall complication rate was 3.3% with no mortalities and no conversions to open or laparoscopic approaches. The overall positive margin rate (PMR) was 11.9%. PMR was 1.4% for pT2a, 0% for pT2b, 8.3% for pT2c, 39.7% for pT3a, and 56.7% for pT3b. Biochemical recurrence rate at one year was 1.7%. Continence rate by 12 months was 94%. Potency rate by 12 months was 83%.

**Conclusions:**

Both perioperative and postoperative outcomes of our series of robotic prostatectomies performed by a single surgeon at Mount Sinai Medical Center demonstrate the superb outcomes that can be achieved through this modality of treatment.

## INTRODUCTION

Radical prostatectomy is considered the ‘gold standard’ treatment for patients with organ-confined prostate cancer and greater than 10 years life expectancy. There are now several surgical approaches available to the urologist: perineal, open retropubic, laparoscopic, and robotic-assisted laparoscopic. Retropubic radical prostatectomy was first reported by Millin in 1947 (1). The surgery was associated with significant morbidity: high blood loss often requiring transfusion, incontinence, impotence, and a prolonged recovery. In the early 1980s Walsh described a new more precise technique of anatomical dissection that improved functional outcomes (2). This became the dominant surgical approach to prostate cancer until the popularity of the robotic approach overtook it in the middle of this decade. Regardless of the type of surgery, the goals of successful radical prostatectomy remain the same: cancer control, urinary continence, and potency.

The advent of laparoscopic surgery and the increasingly popular concept of minimally invasive surgery in the 1990s again brought the next major advance in radical prostatectomy. In 1991 Schuessler (3) performed the first laparoscopic prostatectomy. The technique was refined and popularized by Guilloneau and Vallancien in the late 1990s (4). It has since been demonstrated to be safe, effective, and similar to RRP in oncologic outcomes. Laparoscopic prostatectomy (LRP) provided the benefits of decreased blood loss (secondary to the increased abdominal pressure of the pneumoperitoneum and better visualization) and a minimally invasive approach. However, it remained a technically challenging operation with a steep learning curve and poor ergonomics.

Robotic assisted laparoscopic prostatectomy was first reported by Abbou *et al* (5) in 2000. It was popularized by Menon *et al* (6, 7) as a minimally invasive technique with vastly improved ergonomics and shorter learning curve relative to LRP. In particular, RALP offered a 3-dimensional stereoscopic visualization, intuitive finger-controlled movements with range of motion surpassing that of the human hand. Robotic-assisted laparoscopic prostatectomy continues to gain acceptance and increase in popularity across the world. An estimated 50,000 robotic prostatectomies were performed in 2007 (6); by the end of 2008, this number will likely increase to more than 80,000. Clearly this new technology has become the dominant method of surgical treatment for prostate cancer. As institutions acquire robots and surgeons overcome the learning curve, results continue to improve. There have been reports questioning the merits of this approach (8). However, most series by experienced surgeons report results that are comparable or better than open series. We present our technique and experience of the first 700 cases with robotic assisted laparoscopic prostatectomy at Mount Sinai Medical Center.

## METHODS

Data were collected prospectively in an IRB-approved database from May, 2007 through October, 2008. All surgeries were performed by one surgeon using the same technique (described below). The surgeon had a prior case experience of 510 robotic prostatectomies at another institution prior to this case series.

### Patient Demographics

Patient demographic data is presented in Table 1. There were no patients with Gleason sum 5, which is consistent with the rarity of this pathologic diagnosis. Twenty-four percent of patients had prior abdominal surgery, with hernia repair, cholecystectomy, and appendectomy at rates of 49.7%, 32.1%, and 9.3%, respectively. Eleven patients (0.02%) had undergone prior transurethral prostate surgery for BPH; most of whom had a TURP (0.01%).

**Table 1:** Patient Demographics

Mean Age (years)	59 (40–78)
Mean BMI	27.5 (19–46.9)
Mean PSA	6.0 (0.4–50)
Median Gleason score	6
Gleason score (%)	
6	62
7	31
8	6
9	1
Prior abdominal surgery (%)	24
Hernia repair	49.7
Appendectomy	32.1
Cholecystectomy	9.3
Other	8.8
Prior transurethral prostate surgery	1.6
TURP	1.0
TUNA	0.3
Laser ablation	0.3

### Surgical Technique

The following are the key aspects of our technique: 1) dissection of the bladder neck first; 2) minimal to no use of cautery from posterior bladder neck dissection onward; 3) leaving endopelvic fascia intact until after neurovascular bundles dissected; 4) preservation of a wide margin of endopelvic fascia; 5) posterior dissection and nerve-sparing in a medial to lateral direction; 6) cold transection of the dorsal venous complex without prior ligation; and 7) posterior bladder neck reconstruction.

**Figure 1:**
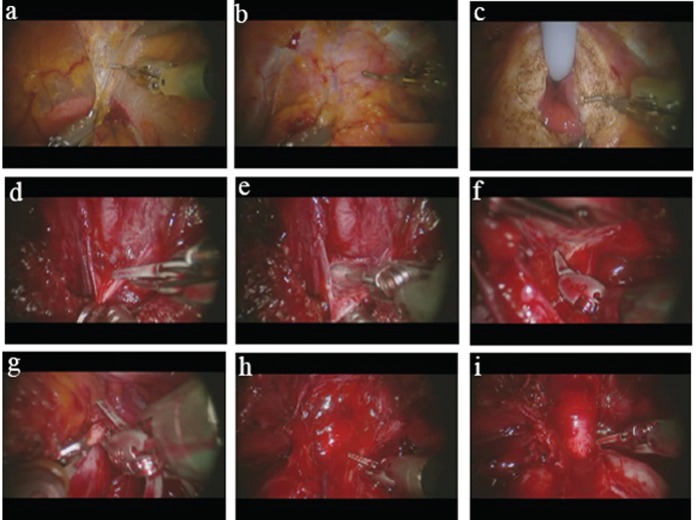
Photographs of selected steps of our robotic prostatectomy technique. (a) Dissection of the space of Retzius using the Maryland bipolar forceps in the left hand and the cautery hook in the right. The bladder can be seen in the bottom portion of the picture beneath the bipolar forceps; (b) The prostate has been cleared of its fatty covering and the anterior bladder neck (being held by the bipolar forceps) is about to be opened with the cautery hook; (c) After the anterior bladder neck has been opened, the Foley catheter is pulled anteriorly with the fourth arm (ProGrasp forceps, not shown in photo), giving exposure to the lateral and posterior portions of the bladder neck; (d) With the prostate lifted anteriorly with the fourth arm, the Denonvillier's fascia is grasped with the bipolar forceps and cut with the cold scissors (bladder neck seen in foreground); (e) The pearlywhite tissue that is characteristic of the plane of tissue between the layers of Denonvillier's fascia is shown here; (f) The neurovascular bundle is gently dissected off the poserolateral surface of the prostate, shown here on the left side (prostate is being held up by left hand bipolar forceps); (g) The endopelvic fascia is opened towards the apex along the contour of the prostate; (h) The dorsal venous complex is divided with the pneumoperitoneum raised to 20 mm Hg; (i) The apex of the prostate is shown here with the urethra skeletonized and about to be divided close the prostate to preserve maximum urethral length.

Briefly, our technique of robotic assisted laparoscopic prostatectomy is as follows: A 4-arm robot is used and a total of 6 ports are placed. After the space of Retzius is developed and the anterior prostate is dissected free of fat (Figure 1a), the anterior bladder neck is opened (Figure 1b), followed by the posterior bladder neck (Figure 1c). After opening Denonvillier's fascia (Figure 1d and Figure 1e), the posterior dissection is carried out in a medial to lateral direction concomitantly with the division of the prostatic pedicles using only hemostatic clips and cold scissors (no cautery). The neurovascular bundles are routinely spared (Figure 1f) unless there is evidence (either preoperatively on MRI or intraoperatively) of extracapsular extension in their vicinity. After the neurovascular bundles are dissected free of the specimen, the endopelvic fascia is divided close to the prostate (Figure 1g), taking care to leave behind as much fascia as oncologically feasible. The apical dissection is then performed. With the pneumoperitoneum raised to 20mm Hg, the dorsal venous complex is cut with cold scissors without prior ligation (Figure 1h). With cephalad traction on the prostate, the urethra and recto-urethralis muscle are then divided close to the prostate (Figure 1i) and the distal end of the dorsal venous complex is oversewn. The posterior bladder neck is reconstructed in a racket-handle fashion. The urethrovesical anastomosis is performed according the standard method (described by van Velthoven *et al* (9)) with two 2-0 monocryl sutures on UR-6 needles tied together at their ends.

### Postoperative course

Patients were maintained on a clear liquid diet until they experienced signs of a return of bowel function, at which point they were advanced to a regular diet. Ambulation was encouraged starting on the day of surgery. Oral narcotics were discouraged but were rarely needed. A complete blood count and metabolic profile was obtained on postoperative day one. Catheters were removed approximately one week after surgery. Patients with very large prostates, median lobes, or high output from the Jackson-Pratt drain had cystograms performed before removing the catheter.

## RESULTS

### Intraoperative Data

Intraoperative data is presented in Table 2. Mean operative time (incision to closure) was 124 minutes [48−266]. Mean robotic time (time spent at the console by the surgeon) was 88 minutes [36−190]. Mean EBL was 69.3 ml [5−400] with 0% intra-operative transfusion rate (two patients required transfusions in the postoperative period: one for anemia secondary to pelvic hemorrhage and one for persistent bleeding from the JP site; total rate of 0.3%). No patient required conversion to open or pure laparoscopic surgery. There was one intraoperative surgical complication – a small bowel injury in a patient with extensive prior abdominal surgery. There were no rectal injuries.

**Table 2:** Intraoperative Data

Mean operative time (min)	124 (48–266)
Mean robotic time (min)	88 (36–190)
Mean estimated blood loss (mL)	69.3 (5–400)
Conversion rate (%)	0
Blood transfusion rate (%)	0
Intraoperative complications	1

### Oncologic Results

Pathologic data is presented in Table 3. Postoperative pathologic analysis showed a mean prostate weight of 53g [22−200]. The distribution of pathologic stages was 11.0% pT2a, 1.25% pT2b, 70.0% pT2c, 11.4% pT3a, and 4.7% pT3b. The median postoperative Gleason score was 7. The distribution of postoperative Gleason scores is as follows: 33.9% Gleason 6, 60.2% Gleason 7, 2.8% Gleason 8, 3.1% Gleason 9. The distribution of positive margin location was as follows: apical 10.3%, base 11.8%, anterior 8.8%, posterior 45.6%, lateral 11.8%, seminal vesicle 10.3%, urethra 1.5%. The positive margin rate for each pathologic stage was as follows: 1.4% pT2a, 0% pT2b, 8.3% pT2c, 39.7% pT3a, 56.7% pT3b. The overall positive margin rate was 11.9%.

**Table 3:** Oncologic Results

Prostate weight	53 (22–200)
Pathologic Stage (%)	
T2a	11.0
T2b	1.25
T2c	70.0
T3a	11.4
T3b	4.7
T4	0
Median Postoperative Gleason score	7
Postoperative Gleason score (%)	
6	33.9
7	60.2
8	2.8
9	3.12
Positive surgical margins by location (% of total positive margins)	
Apical	10.3
Base	11.8
Anterior	8.8
Posterior	45.6
Lateral	11.8
Seminal vesicle	10.3
Urethra	1.5
Positive surgical margin rate by stage (%)	
pT2a	1.4
pT2b	0
pT2c	8.3
pT3a	39.7
pT3b	56.7
Overall positive surgical margin rate (%)	11.9
Biochemical recurrence rate (%)	1.7

Also shown in Table 3 is the biochemical recurrence rate of 1.7%. These patients had a PSA ≥0.2 at 3 months or greater (with up to 12 months follow-up; n=530).

### Postoperative Data and Complications

Mean and median length of hospital stay was 1 day. Mean catheter time was 7 days (4–30). As outlined in Table 4, total number of complications was 20 (3.3%), consisting of 3 cases of pulmonary emboli, 2 DVTs, 1 pelvic hemorrhage requiring transfusion, 4 episodes of urinary retention, one port site hernia (which required a return to the operating room for repair), 2 ileus, 1 bowel obstruction, 1 lymphocele, 3 prolonged hematuria, 1 epididymo-orchitis, 1 JP site hemorrhage requiring transfusion, 4 pelvic collections, 1 bladder neck contracture, one wound infection, and one anastomotic leak.

**Table 4:** Perioperative and Postoperative Complications (number of complications)

Pulmonary embolus	3
DVT	2
Urinary retention	4
Port site hernia	1
Ileus	2
Bowel obstruction	1
Small bowel injury	1
Rectal injury	0
Lymphocele	1
Prolonged hematuria	3
Epididymo-orchitis	1
Pelvic hematoma requiring transfusion	1
JP site bleeding (requiring transfusion)	1
Pelvic collection	4
Bladder neck contracture	1
Wound infection	1
Anastomotic leak	1
Overall complication rate (%)	3.3

### Quality of Life Outcomes

**Figure 2:**
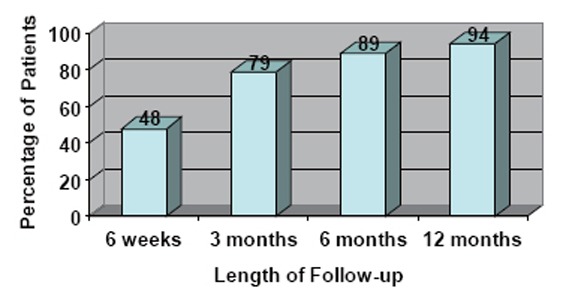
Postoperative continence rates (percentage of patients with 0–1 security pad per day) at demonstrated intervals up to one year.

Continence was defined as 0–1 pads/day, where one pad is for security or mild occasional stress incontinence. Continence outcomes for patients who were preoperatively incontinent or had a AUA symptom score >20 were omitted from analysis. There were 422 patients with follow-up data. Figure 2 demonstrates postoperative continence rates at 6 weeks, 3 months, 6 months, and 12 months follow-up.

**Figure 3:**
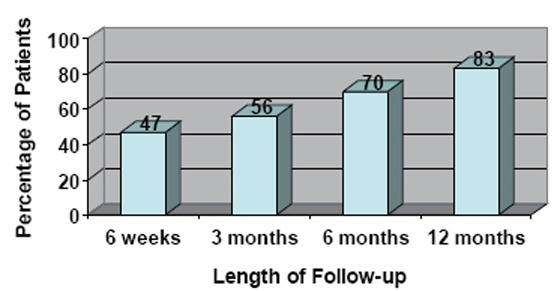
Postoperative potency rates (percentage of patients with SHIM score >21 or ability to have intercourse) at demonstrated intervals up to one year.

Potency was defined as the ability to have sexual intercourse and/or SHIM (Sexual Health Inventory for Men; a validated questionnaire assessing erectile dysfunction on the basis of a 25-point score) score of 21 or greater. Potency outcomes are reported only for those patients who were potent prior to surgery and for those who underwent bilateral nerve-sparing prostatectomy. There were 309 patients with follow-up data. Figure 3 demonstrates potency rates at post-operative intervals of 6 weeks, 3 months, 6 months, and 12 months.

## DISCUSSION

Robotic radical prostatectomy continues to evolve at a rapid pace. As robotic surgeons increase their cumulative volume of cases, refinements continue to be made in order to improve outcomes. A search for ‘robotic prostatectomy’ on PubMed will find several new articles on technique appearing every month. The rationale for perfecting the surgical technique of robotic radical prostatectomy is the enormous potential of the robotic approach. The magnification and manual dexterity offered by the robot allows a higher level of attention to detail and resultant precision in surgery. We believe that there are certain salient features of robotic technique that impact on the quality of the surgery and its oncologic and functional outcomes. Every step of the operation is undertaken with the overarching aim of completely excising the cancerous prostate while preserving the surrounding normal tissues.

One of the goals of our robotic technique is maximal preservation of the endopelvic fascia surrounding the prostate. Recent studies support the theory that preservation of the endopelvic fascia improves functional outcomes. Indeed, van der Poel *et al* (10) recently demonstrated that fascia preservation at the lateral aspect of the prostate was a strong predictor of urinary continence post-RALP. In their analysis, a quantitative scoring system was used to assess the extent of circumferential fascia preservation. They hypothesized that lateral nerve bundles contained within the lateral fascia are better preserved when more fascia is left behind. A similar improvement in potency outcomes was noted as well with more extensive lateral fascia preservation (11).

Another important aspect of technique which likely affects all three outcomes (cancer control, continence, and potency) is the control and division of the dorsal venous complex (DVC). We find that omitting the DVC stitch and cutting with the cold scissors with simultaneous cephalad traction on the prostate allows for a more precise division of both DVC and urethra. There are several implications of this technique. Most importantly, a stitch distorts the anatomy and dictates where the surgeon will divide the apex, potentially causing a positive apical margin. Guru *et al*. (12) recently demonstrated a statistically significant decrease in positive apical margin rate when performing a cold incision of the DVC before suture ligation. The stitch also can potentially trap the nerves which partially run alongside it. Innervation of the urethral sphincter is closely related to the prostatic apex (13), and men with post-prostatectomy incontinence have been demonstrated to have decreased function of these nerves (14). Finally, because of the superb visualization of the prostatic apex and reduced bleeding from the dorsal vein complex (secondary to pneumoperitoneum), a more precise incision of the urethra is possible, allowing a longer urethral stump to be achieved. Preoperative urethral length assessed by magnetic resonance imaging (MRI) has predicted earlier continence recovery (15, 16) with increasing urethral length.

Our oncological results are on par with those reported elsewhere in the literature for robotic prostatectomy (17). Our overall positive margin rate was 11.9%. When subdivided by pathologic stage, the margin rate increased with more extensive disease, as expected (39.7% and 56.7% for pT3a and pT3b, respectively). Almost half of our positive margins were located posteriorly, with almost equal lower rates at other locations. This may be explained by the aggressive nerve-sparing approach, whereby the dissection often proceeds very close to the capsule while dissecting posteriorly and posterolaterally. The comparatively low apical margin rate is likely due to the superb visualization offered by the robotic technique as well as the omission of the dorsal vein ligation prior to transection. As explained earlier, the dorsal vein stitch may distort the anatomy of the apex and thereby lead to cutting into the wrong plane.

Our continence and potency rates (94% and 83%, respectively) at 12 months compare favorably to rates reported elsewhere (7, 18–23) (Table 5). In summary, with regards to preservation of continence, our experience has shown that adherence to the following principles yields the best results: 1) no cautery on the urethra; 2) preservation of periurethral musculature, including intrinsic rhabdo-sphincter and puboperinealis; 3) avoidance of hemostatic sutures in the dorsal vein complex, which potentially could damage the sphincter; 4) preservation of maximum urethral length; and 5) a precise water-tight anastomosis with posterior bladder neck reconstruction. In addition to avoiding direct local damage to the external urethral sphincter as a way to preserve continence, lateral dissection of the urethra distally (near the external striated sphincter) also should be avoided as it risks damage to both the pudendal and pelvic nerve branches to the urethra. With the superb visualization offered by the robot, the posterolateral prostate is readily visualized and the proper plane of dissection between the neurovascular bundle and the prostatic capsule may be more readily identifiable and precisely incised. Key aspects of our technique aimed at preserving sexual function include: 1) preserving the neurovascular plate (defined by Tewari *et al* (24)) during dissection of bladder neck and seminal vesicles; 2) partially releasing the neurovascular bundle before pedicle control; 3) incising the lateral prostatic fascia parallel to neurovascular bundle, following the configuration of prostate to release the neurovascular bundle (leaving behind a wide margin of fascia); and 4) developing the prerectal space and exposing the neurovascular triangle.

**Table 5:** Summary of continence and potency outcomes at one year for selected series reported in the literature

Author	Continence (≤1 pad/day) (%)	Potency (%)

Menon *et al* (7)	95.2	70
Joseph *et al* (18)	96	70
Patel *et al* (19)	95	78
Zorn *et al* (20)	90.2	80
Badani *et al* (21)	93	79.2
Mottrie *et al* (22)	95	70
Murphy *et al* (23)	91.4	64

It is also important to take into account the experience of the robotic surgeon. In this case, the surgeon had performed 510 robotic prostatectomies at another institution prior to this series. He also had extensive experience with the pure laparoscopic prostatectomy technique as well as open surgery. Training in open and pure laparoscopic surgery provides a strong framework upon which to build a superior robotic technique. Our data show a low complication rate of 3.3%, which compares favorably to most open or laparoscopic series. The most common complications were pelvic collections and urinary retention, followed by pulmonary embolus and prolonged hematuria. Of note, there were no rectal injuries and only one small bowel injury in a patient with prior bowel surgery.

Downstaging of disease in the modern PSA era has created a patient population consisting of mostly low to intermediate risk candidates for surgery. This allows for a ‘less is more’ approach in operating, which is now more possible than ever due to the capabilities of the robot. Tactile feedback has been replaced by enhanced visualization in a bloodless field. The overarching aim is to stay away as much as possible from the lateral tissues to avoid damage to important structures. The surrounding fascial structures are spared as much as possible, preserving the maximum allowable length of urethra.

Risk of injury to the neurovascular bundles in the region of the apex of the prostate can be minimized in robotic surgery by performing a thorough apical dissection before dividing the DVC and urethra.

Current robotic techniques represent a natural extension of open techniques. For example, modifications in open surgical techniques, such as placing a 22-gauge surgical wire beneath the DVC (anterior to the urethra and just distal to the prostatic apex) were aimed at increasing the accuracy of division of the DVC. Now, aided by 3-dimensional, magnified vision, the robotic surgeon can use cold scissors while retracting the prostate cephalad (as one would have done with a sponge stick in open surgery) and accomplish a precise and accurate division of the DVC without the added step of placing a stitch or wire. We also believe that an antegrade approach to nerve dissection (dissecting in a medial to lateral direction) is less traumatic than the early retrograde release (starting laterally with initial opening of the endopelvic fascia) of the neurovascular bundles. Other approaches to the DVC which have been described, such as control with the endo GIA stapler or a suture ligature, are both unnecessary and potentially detrimental to the results.

## CONCLUSIONS

As critics appropriately point out, the robot does not automatically turn any open surgeon into a better prostatectomist. The enhanced capabilities of the robot allow for potentially superior surgical techniques, but these techniques require skill and practice on the part of the surgeon. For example, an inexperienced robotic surgeon can potentially cause an unnecessarily high rate of positive surgical margins during such a close dissection off of the prostatic capsule. However, with time the surgeon will learn the visual cues that aid in avoiding this pitfall.

Never before have surgeons enjoyed the enhanced visualization, flexibility, and dexterity that the robot offers. Though on the whole up to this point, RALP outcomes have essentially been similar to those of open, we believe that they will soon surpass them. Our series of 700 consecutive cases performed by a single surgeon demonstrates the excellent perioperative and postoperative outcomes which are representative of high-volume surgeons.
